# The next evolutionary synthesis: from Lamarck and Darwin to genomic variation and systems biology

**DOI:** 10.1186/1478-811X-9-30

**Published:** 2011-11-03

**Authors:** Jonathan BL Bard

**Affiliations:** 1 Department of Physiology, Anatomy & Genetics, University of Oxford, UK

## Abstract

The evolutionary synthesis, the standard 20^th ^century view of how evolutionary change occurs, is based on selection, heritable phenotypic variation and a very simple view of genes. It is therefore unable to incorporate two key aspects of modern molecular knowledge: first is the richness of genomic variation, so much more complicated than simple mutation, and second is the opaque relationship between the genotype and its resulting phenotype. Two new and important books shed some light on how we should view evolutionary change now. *Evolution: a view from the 21^st ^century *by J.A. Shapiro (2011, FT Press Science, New Jersey, USA. pp. 246.) examines the richness of genomic variation and its implications. *Transformations of Lamarckism: from Subtle Fluids to Molecular Biology *edited by S.B. Gissis & E. Jablonka (2011, MIT Press, Cambridge, USA. pp. 457) includes some 40 papers that anyone with an interest in the history of evolutionary thought and the relationship between the environment and the genome will want to read. This review discusses both books within the context of contemporary evolutionary thinking and points out that neither really comes to terms with today's key systems-biology question: how does mutation-induced variation in a molecular network generate variation in the resulting phenotype?

## Introduction

In the latter half of the twentieth century, the view of the process by which new species originated was based on meshing Darwinian variation and selection with work on population genetics and mutation. This view, the *evolutionary synthesis*, suggested that mutation within individuals led to genetic variation within a population; should a subgroup of that population become genetically isolated in a novel environment, a mix of unbalanced variation and new mutation within the subgroup would lead to new phenotypes appearing. In due course, one of these might reproduce better than the original phenotype so that it would take over and eventually lead to the appearance of a new species with a novel genotype [1].

The enormous amount of molecular information that has emerged during the last couple of decades is making us review this synthesis, partly because we now know that the relationship between the phenotype and genotype is not as simple as previously assumed, partly because the genome is a richer, more complicated world than the scientists who put together the modern synthesis could ever have supposed and partly because there is data that does not fit comfortably within the synthesis. The two interesting and important books under review here set out to examine aspects of the state of evolutionary science now, the one taking an unashamedly contemporary position, the other starting from 200 years ago. Before discussing what they have to say, it is worth taking a look at their context by considering the state of evolutionary biology today.

The evidence for evolution itself is robust as it comes from the three independent lines that each tells the same story: history (fossil record and isotope dating), morphology (taxonomic relationship and comparative embryology in living organisms - evolutionary change starts off as developmental change) and molecular sequence relationships. While the evolutionary synthesis is of course compatible with evolution, the evidence to support it is actually much thinner than is generally supposed; this is mainly because data is hard to come by in processes that are intrinsically slow and rare. One line of supporting evidence is the existence of ring species such as the various greenish warblers around the Himalayas where neighbouring subspecies around the ring can interbreed, but there is a break point where the two adjacent ones, although members of the same family, cannot and so have to be viewed as separate species [2]. A line of work that the evolutionary synthesis cannot explain so easily is Waddington's remarkable set of experiments on selection [3]. The most famous of these involved first making a phenocopy of the *bithorax *mutation (a four-winged fly instead of one with two wings and two halteres) by ether treatment of wild-type flies and then interbreeding these phenocopies under strong selection (rejecting all offspring that didn't produce the ether phenocopy). He found that, after ~20 generations of interbreeding and selection, he had a population where the four-winged flies bred true without further ether treatment. Waddington called this process, which was too fast to be initiated by novel mutations, *genetic assimilation*.

There is a serious underlying problem with the evolutionary synthesis: it is based on a minimalist Mendelian view of genetics which assumes that a very small number of genes underpin a trait and a mutant gene leads to an abnormal phenotype. While the advantage of the formulation is that it provides a model for evolutionary genetics [4], the disadvantage is that the approach assumes a naively simplistic view of how genes generate traits, as Waddington pointed out in the '50s [5]. If more than about three genes (nature unspecified) underpin a phenotype, the mathematics of population genetics, while qualitatively analyzable, requires too many unknown parameters to make quantitatively testable predictions [6]. The inadequacy of this approach is demonstrated by illustrations of the molecular pathways that generates traits [7]: the network underpinning something as simple as growth may have forty or fifty participating proteins whose production involves perhaps twice as many DNA sequences, if one includes enhancers, splice variants etc. Theoretical genetics simply cannot handle this level of complexity, let alone analyse the effects of mutation.

We now know that there are at least 50 possible functions that DNA sequences can fulfill [8], that the networks for traits require many proteins and that they allow for considerable redundancy [9]. The reality is that the evolutionary synthesis says nothing about any of this; for all its claim of being grounded in DNA and mutation, it is actually a theory based on phenotypic traits. This is not to say that the evolutionary synthesis is wrong, but that it is inadequate - it is really only half a theory! Much as classical thermodynamics needed statistical mechanics to provide a theory of heat and work based on molecular physics, so the evolutionary synthesis needs to incorporate a proper model of DNA variation and a more sophisticated means of linking phenotypes to genotypes than Mendelian genetics. A modern version of the evolutionary synthesis thus has to be based on the reality of the genome and how it works; in particular, it has to provide answers to three key questions about how organisms change.

How has genetic variation generated contemporary organism diversity and complexity from simple beginnings?

While the story of evolution is qualitatively different from that of the origin of life, it is important to distinguish between the origins of simple bacteria and of multicellular organisms that show cellular differentiation. The former is lost in the mist of time but it is remarkable that, in the comparatively short time between the appearance of Ediacaran organisms (c. 620 MYA) and those of the lower Cambrian (c. 530 MYA), a large number of novel cell types and tissue morphologies evolved, as can be seen in some of the early soft-bodied fossils such as Haikouella [10] This part of the origin of life should be within the remit of a good modern theory of evolution.

What controls the rate of evolution?

This area currently generates more heat than light! Selection pressure is part of the story but we do need some insight into why evolution sometimes seems to go very rapidly.

How is genetic variation manifested as phenotypic variation?

This last question meshes with a key problem of contemporary systems biology, one of whose aims is to work out how complex networks of proteins generate developmental and physiological functions and how mutation affects output.

## The importance of generating genomic variation

We know where we want to be and the two books under review reflect on how far along the road we are. Shapiro's *Evolution, a view from the 21^st ^century *(2011, FT Press Science, New Jersey, USA. pp. 246. $34.99) takes a superficially straightforward view of things by focusing on the first of the questions: he takes the view that, once the genetic variation is in place, the rest of evolutionary process inevitably follows so that the problem now is to understand the evolution of DNA so that it can be grafted onto the evolutionary synthesis. There is therefore very little on genotypes, selection, genetics or speciation in the four chapters of text that comprise 50% of a book of almost 250 pages. The rest is tables, a glossary, an index and 65 pages of references; these are also in the online appendices together with thousands of additional references, many of which are linked to the papers in Pubmed (this considerable service to the community must have taken weeks of tedious work). By focusing on variation, Shapiro sets out to update the 20^th ^century synthesis of evolution to the modern synthesis of the 21^st ^century by showing how it needs to come to terms with the richness of genomic complexity.

The book is thus situated within the general context of systems biology and bioinformatics in the sense of the informatics of biology rather than its usual meaning (computational sequence analysis), it is thus a refreshingly modern read. The heart of the book is its analysis of the diversity of genetic sequences and how these can be generated, with every fact supported by references. I learnt a lot both from reading the text and from being forced to look at the genome in a way that I had previously been too lazy to approach on my own. I also appreciated the lengthy tables that list examples across the phyla to buttress more general points about mechanisms. This is an important book that anyone interested in the process of evolutionary change should read, and I suspect that many future papers will be built on the foundations that it provides.

That said, the book has some limitations that will, I hope, be dealt with in the further editions to be expected in an area where the *zeitgeist *keeps moving. The minor problems are stylistic. First, the author writes as if he is giving a lecture to students and, while I am comfortable with bullet points, I am less so with being addressed as "you". To appreciate this book, the reader really needs at least degree in modern biology and the style is slightly patronizing. The author's hope that the amateur will get something from a book as technical as this is wishful thinking. Second, and more important, the absence of diagrams is surprising in the book, and this is particularly inappropriate given that the author describes the many and complicated ways in which DNA sequences can be used and changed, an area that lends itself to graphical explication. There are, it should be said, some diagrams in the online appendices, but these are not helpful in following the text. Third, there are one or two careless sentences that need to be removed. On page 95, Shapiro writes "eukaryotes progressed from yeast through nematode worms and *Drosophila *fruit flies to mice and human beings" and I really don't think that he believes in the 18^th ^century view that evolution progressed up the ladder of life (see below). The pedant might also take issue with his criticism of Darwin (page 121) for postulating that selection never gave rise to new species - new species never arise without a degree of selection and the distinction between mechanisms being permissive and directive was not as clear in the 19^th ^century as it is today.

More important are the scientific limitations. First, while Shapiro details the ways in which DNA can be expanded, duplicated, altered and mutated, he pays little attention either to how changes in an individual can be assimilated within a population or to how these changes can alter the downstream genotype and so be subject to selection. Second, a disproportionate amount of the text is given to the genomes of bacteria and other unicellular organisms as opposed to those of animals and plants where the importance of variation in germline cells is barely mentioned. Perhaps because the author is a bacterial geneticist, he does not use this information to discuss the evolution of these more advanced organisms and the book feels a little unbalanced. Third, and this brings me to the most serious criticism of the book: evolution means change over time and time barely merits a mention. Shapiro lists the ways that DNA can alter, but barely discusses whether, when, where and how the various mechanisms of genomic variation might have been used. There is now enough sequence data available to analyze the various amplifications, duplications, insertions and other high-level changes within the evolutionary DNA hierarchy to allow us to see when and where they occurred and what role they had (e.g. the Hox gene set whose evolution has been studied by Peter Holland and his colleagues [11]). My gut feeling is that the much of the richness was needed to generate the genotype richness that characterized the evolution of simple multicellular organisms to the highly differentiated ones that characterized the Cambrian fauna. Either way, the next edition will need a new chapter that will consider when and where the various types of variation were used and to what purpose.

## Lamarckian evolution

Given Shapiro's focus on the generation of genomic variety, it is perhaps surprising that he does not discuss the possibility, even to dismiss it, of there being some feedback from an organism's response to a novel environmental to the germline so that it might be passed on to successive generations. This idea, first formally articulated by Lamarck in 1809 [12] and believed by Darwin who provided a mechanism to achieve it that he called pangenesis [13], has had a bad press for two centuries because we have no accepted mechanism to achieve it, other than Waddington's genetic assimilation and that is controversial in this context. Nevertheless, the idea won't go away partly because organisms are so well tuned to their environment and partly because standard genetic change seems too slow to explain how this occurs in a reasonable time (it was no coincidence that chapter 1 of *The origin of species *discusses rapid variation in pigeons [14]). As 2009 was the 200^th ^anniversary of the publication of Lamarck's *Philosophie Zoologique*, it seemed sensible to organize a meeting in Jerusalem to celebrate his work and to explore the validity of his ideas today and publish the papers in *Transformations of Lamarckism: from Subtle Fluids to Molecular Biology *(2011, edited by S.B. Gissis & E. Jablonka, MIT Press, Cambridge, USA. pp. 457. $50.00).

Lamarck, it should be said, was the foremost invertebrate zoologist of his generation: he was the professor of invertebrate zoology (insects and worms) at the Muséum national d'Histoire naturelle in Paris (1793 - c.1820), he introduced the words "biology" and "invertebrate", articulated the difference between homologous and analogous adaptations and wrote many books on botany, invertebrates and evolution. Insofar as there was a theory of evolution at the turn of the 19^th ^century, it was Bonnet's idea [15] that evolution involved climbing the ladder of complexity to its top rung of man. Lamarck showed that this idea had to be wrong when he realized that it was not geometrically possible for annelid worms to have evolved from parasitic worms and both must therefore evolved from another type of organism. Lamarck thus showed, and he was the first to do so, that evolution required branching descent (see Gould [16]). Lamarck had a rich academic career, but was not an easy person and made an enemy of Cuvier who wrote a damning obituary dismissing him as an impractical theoretician. Although Cuvier was considered unfair then, it is his view that is accepted now and the time is ripe, as indeed it always has been, for Lamarck's good name to be restored. He was a very clever and thoughtful man who was the first modern evolutionary biologist, as Darwin indeed acknowledged, albeit not until the 6^th ^edition of *The origin of species *[14].

Lamarck's bad reputation is based solely on his ideas about the process of evolution and it is worth spending a moment on what these actually were. Building on general ideas around at the time, he suggested [12] that animals had two intrinsic abilities that allowed them to vary and hence allow evolution to take place. First, there was the *Le pouvoir de vie *- the force that led to increasing complexity and was responsible for the evolution of the major life forms (hence *homologous *structures); second, there was *L'influence des circonstances *- the adaptive force that was responsible for adaptation of animals to their environment (and *analogous *structures). In this context, he suggested that use or disuse of a characteristic led to its progressive inheritance or loss and this, in turn, implied the inheritance of acquired characteristics, albeit with no clear mechanism as to how these characteristics were acquired. While it is claimed that Lamarck meant that it was mental effort that led to change (the giraffe stretching its neck to feed on treetop leaves so that the next generation inherited a longer neck), it is not clear that this was the only idea that he had in mind - Lamarck's language sometimes lacked the clarity that we expect in scientific writing today.

Considering that Lamarck was feeling his way in a new area of biology and working alone more than 50 years before Darwin and Wallace, his analysis was impressive and no one is concerned about the first of his abilities (*Le pouvoir de vie*). It is the second, on adaptation, that is problematic. It is generally considered that direct and rapid feedback from the environment to the germline, or *soft inheritance *to use Darlington's and Mayr's phrase [17,1], cannot happen. There are two obvious ways, in principle at least, in which this can occur. The first is that there is some mechanism, as yet unknown, by which an unusual physiological response by an organism to a novel environment leads to some germline change. The second is that some individual in the population not only develops some variant in its phenotype that allows it to do well in that novel environment but that this variant is heritable. The first says that heritability follows physiological novelty; the second says that a new, immediately heritable change allows that novelty. We do not yet seem to have a mechanism for the former, but, as argued below, a systems approach suggests that the latter might occur more easily than is generally thought. It should be said that that line of thought is not the focus of *Transformations of Lamarckism*.

This impressive book includes some 40 papers organized by themes (history, the modern synthesis, biology, philosophy and ramifications and future directions); all are worth reading although the typical reviewer might wish that they had had abstracts. While I will concentrate here on those papers in the biology section, I would like to say that the book is a beautifully produced and an absorbing read; it should be on the shelf of any biologist with a serious interest in evolution. The historical essays are fascinating, the discussions of the development of the modern synthesis and why the ideas of *soft inheritance *have fallen out of favour [Wilkins] give an important perspective on 20^th ^century work on evolution, while the essays on the wider implications of evolution today should be enjoyed by anyone interested in the wider themes of biology. The two edited reports (so much tauter than verbatim ones) of the discussions are worth reading because they include so many interesting experiments. The editors are to be congratulated for producing a book that should do much to restore the good name of Jean-Baptiste Lamarck.

There are two areas that the book considers that are relevant to a technical discussion of contemporary evolutionary biology in the context of Lamarckian thinking: those data that fit more comfortably within a Lamarckian than a Darwinian framework, and the work on how germline DNA can be altered as a response to environmental change. Theses topics are the focus of the *Biology *section of the book and its chapters and the associated discussion cover almost a lecture course on non-Mendelian inheritance. Of particular note are Markel & Trut's description of the Russian experiments on the domestication of wolves, Braun & David's extraordinary results on how *Saccharomyces cerevisiae *adapts to genomic rewiring, and the work on genetic assimilation in butterflies and in *Arabidopsis thaliana *that are brought up in the discussion.

The strongest reason for rejecting Lamarckian thinking or soft inheritance is the difficulty in providing a mechanism for genomic change that is a response to environmental pressure. The chapter and appendix by Jablonka summarises, within a historical context, much of the work now being done on the many known examples of epigenetic inheritance through methylation, chromatin-marking, RNA-mediated inheritance and structural templating. These ideas are discussed in detail in the following chapters and it is clear that plants are rather easier model systems to work with here than animals partly because of the speed of change [Feldman & Levy] and that there is a great deal more to be learnt from symbionts [Gilbert]. If there is a conclusion, it is that we cannot yet reject the idea that germline change can occur in ways other than through standard mutation.

The key feature of Lamarckian evolution that distinguishes it from the evolutionary synthesis is speed: it requires that phenotypic change be assimilated *rapidly *within a population through selection. Both of these books in their different ways touch on this problem, albeit lightly: Shapiro sees it as deriving from major genomic change, but, while he provides example of how this might happen, does not consider either how this might lead to a change in phenotype or how it might be assimilated. The book on Lamarck considers how an organism's response to a novel environment might lead to genomic change through selection and, as just discussed, points to possible examples. Neither really faces up to how such change can be achieved rapidly, and this is because neither properly tackles the question of the relationship between genotype and phenotype, and how changes in the former lead to changes in the latter.

This problem is at the heart of contemporary systems biology: we now know the details of the many regulatory gene-protein networks responsible for generating a phenotype (growth, pigmentation etc. [7]), but have no theory for predicting their outputs. Worse, natural variation means that there is a spread in the quantitative properties in these outputs (e.g. human face variation derives from minor changes in the local growth pathways and these depend on the rate constants of the interactions which in turn depend on the details of protein structures). Because of the complexity of the networks, the effect of mixing genomes through sexual reproduction and the chance of mutation, it seems unlikely that we will ever be able to predict the details of network output in an organism. Nevertheless, this richness carries an interesting implication.

Complex systems have properties that cannot be predicted, albeit that they can be understood with hindsight, and it may well be that the network for some trait (e.g. bone growth or pigmentation pattern) in the offspring has quantitative properties that are very different from those of the parents, not because of new mutations but because the novel mix of the rate constants will yield a trait that is an outlier of the normal distribution (known as a *sport *in breeding circles). As a result, the offspring may be able to colonise a novel environment far better than its peers. Equally important, this variant will naturally be heritable because it derives from the kinetics of the network (minor variation) rather than additions or losses to the proteins that comprise them [18]. Such immediate heritability within the population is the key requirement for Lamarckian purposes, as Waddington pointed out half a century ago [19]. If this view is correct, it becomes easy to see how something indistinguishable from Lamarckian inheritance can take place.

It is one thing to suggest that this is how evolution can work rapidly in a way that would be seen as Lamarckian, but it is another thing to demonstrate that it is so. As of now, we have no good theory of how to read networks, how to model them mathematically or how one network meshes with another; worse, we have no obvious experimental lines of investigation for studying these areas. There is a great deal for systems biology to do in order to produce a full explanation of how genotypes generate phenotypes and so provide the basis for a full 21^st ^century model of evolution. As T.S. Eliot almost said: "Between the phenotype and the genotype falls the shadow".

## Competing interests

The authors declare that they have no competing interests.
